# The National Rehabilitation Information Center (NARIC)

**DOI:** 10.5195/jmla.2022.1515

**Published:** 2022-07-01

**Authors:** Marilia Y. Antunez

**Affiliations:** 1 Life & Allied Health Sciences Librarian, Associate Professor, Bierce Library, The University of Akron, Akron, OH.

## Abstract

**The National Rehabilitation Information Center (NARIC).** 8400 Corporate Drive, Suite 500, Landover, MD 20785; https://naric.com/; free.

The National Rehabilitation Information Center (NARIC) is an authoritative information resource designed to serve the diverse information needs of consumers, researchers, and professionals interested in disability, independent living, and rehabilitation related topics. As the research center and library for the National Institute on Disability, Independent Living, and Rehabilitation Research (NIDILRR), NARIC's mission is to collect, catalog, and disseminate the results of research produced by NIDILRR grantees and contractors, other US federally- funded projects, and relevant resources including international and commercially published books, journal articles, and multimedia items [1]. NARIC is sponsored by NIDILRR, the US federal government's primary disability research organization and it is operated by HeiTech Services, Inc., under a federal contract. It was originally funded in 1977 by the U.S. Department of Education [2].

NARIC is one of the most comprehensive online resources in the areas of rehabilitation and disability that would complement a search for different levels of users. NARIC consists of four collections: the REHABDATA database and the NIDILRR Program Database; and two smaller collections of organizations, agencies, and online resources (the Knowledgebase), and miscellaneous consumer-oriented information and other relevant resources. NARIC covers a wide range of issues (e.g., assistive technology, special education), and provides current records to different publication types from directory information to evidence-based resources. When searching for research articles published in scholarly journals, users should begin their search in the REHABDATA, the flagship bibliographic database from NARIC. However, it is warranted to search NARIC collections as a whole for a thorough review and to locate additional key resources and specialized resources not found in major subscription databases.

## COVERAGE

As of December 2021, NARIC's three databases (REHABDATA, NIDILRR Program Database, and Knowledge-base) contained more than 339,000 records dating from 1956 [3]. In general, NARIC adds 3,000-5,000 new records per year. Updates to the content often depends on how many documents NARIC receives from journals and from NIDILRR grantees [4]. The date of when a record is loaded into NARIC is reported on each NARIC record. Unique to NARIC is the original research produced by NIDILRR grantees that is not available in other resources.

The core content in NARIC is in its REHABDATA database. Users will find a compilation of over 150 peer-reviewed and scholarly journals, magazines, and newsletters, as well as material from more than 3,000 research projects funded by NIDILRR over its more than 40 years history [5]. In addition to peer-reviewed journal articles, NARIC's specialized collections contain citations to other grey literature such as technical reports, newsletters, brochures, guides, curricula, and fact-sheets. Audiovisual materials (e.g., streaming videos, and webcasts) can be searched in the NARIC Multimedia Collection from the NIDILRR community/affiliates [6]. Some of NARIC's citations to international documents were absorbed from the Center for International Rehabilitation Research (CIRRIE), a retired database of international rehabilitation research [7,8]. Non-English language publications such as selective Spanish-language consumer-oriented materials are also available.

As a free index, NARIC's access to full-text articles from licensed journals, books, or book chapters is limited, and records do not include stable links. When full text is available, the PDF is either included at the top of the record, or there is a hyperlink to the full text.

The document delivery service link is conveniently included in each NARIC record. Full-text articles may be ordered through NARIC for a small fee, and the paper copy is sent via regular mail.

## SEARCH

**Figure 1:**
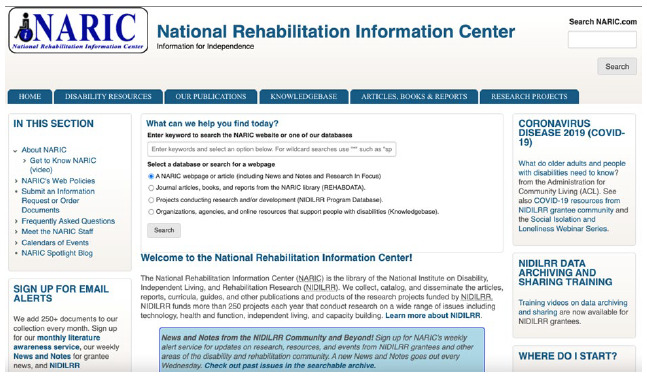
Top part of the NARIC home page, “Home” tab.

Basic keyword searching on a single search box is available in the “Home” tab, but users must select one specific collection (See Figure 1).

**Figure 2:**
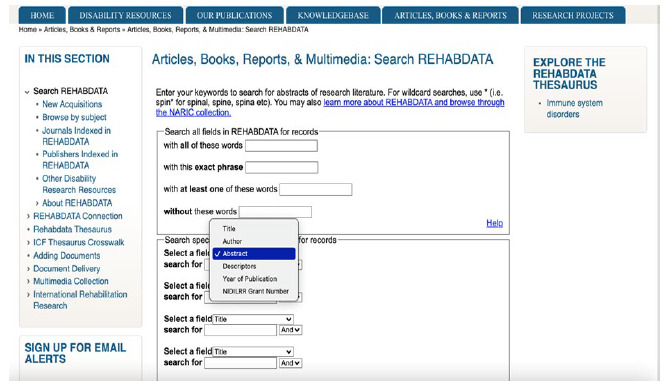
Filter options in the advanced search in REHABDATA, “Articles, Books, Reports, & Multimedia” tab.

The Boolean operator AND is not required when doing a search. A “Search Naric.com” search box is located at the top right of every NARIC webpage and searches the entire NARIC collections, but the search results retrieved are not organized. The advanced search screen is available only when separately searching the NIDILRR Program Database, REHAB-DATA, or Knowledgebase databases. Boolean operators are described and are available to use but are not displayed (e.g., “with all of these words” searching field represents the AND operator). Truncation and phrase searching are allowed. Users can limit a search by the Thesaurus term (called Descriptors in NARIC) in the REHAB-DATA only and use other filters (See Figure 2). A search history is available (i.e., called Previous Searches).

REHABDATA, NIDILRR Program Database, and the Knowledgebase databases are browsable and use the REHABDATA Thesaurus to index research and reference collections. The thesaurus can also be used to retrieve other information collected by NARIC because it was used to organize NARIC's navigation system [9,10]. The thesaurus terms may be browsed but they may not be searched by a keyword search. These discipline-specific thesauri are hyperlinked and are listed in alphabetical order [11]. The REHAB-DATA Thesaurus was developed by NARIC and is regularly updated by disability and rehabilitation researchers and professionals [12].

Search results are displayed in a brief format and include a hyperlinked project number or an accession number that can direct users to the detailed NARIC record. Twenty search results are displayed in chronological order with the current records listed first. Each record includes subject headings. For all or selected records, users have the option to create a bibliography, and print or export records in XML.

## FORMAT AND ORGANIZATION

The NARIC platform contains six tabs or webpages: “Home”; “Disability Resources”; “Our Publications”; “Knowledgebase”; “Articles, Books & Reports”; and “Research Projects” (Figure 1). The REHABDATA database is searchable from either the home page (i.e., “Home” tab) or from the “Article, Books & Reports” tab. In the NIDILRR Program Database (i.e., the “Research Projects” tab), users can search for records of completed and ongoing research projects funded by NIDILRR. Each record includes an abstract.

The Knowledgebase (i.e., the “Knowledgebase” tab) is a searchable collection and provides detailed records of individual organizations, agencies, publishers, databases, and other online resources at local, state, and national level organizations. The Knowledgebase is a convenient way to locate these relevant organizations that provide support to consumers.

The two remaining curated and browsable collections provide ready reference-type resources, research tools, and consumer information materials to help address common questions such as those about treatment, benefits, and services. These resources are highlighted in the “Disabilities Resources” and the “Other Resources” tabs. In the “Disabilities Resources” tab, users will find a selected list of twenty subjects (e.g., Resources for Specific Disabilities) with links to contact information for relevant organizations, and other online resources. Similarly, in the “Our Publications” tab, users will find a hyperlinked list of recommended resources (i.e., Librarian's Picks), grant opportunities and information, research funding, and other NIDILRR resources (e.g., The Life Skills Manual: Strategies for Maintaining Residential Stability). Librarians may want to use these collections to introduce their users to key US organizations. Noteworthy resources for researchers include NARIC's collection of more than 40 research instruments, some available in full text; the NIDILRR Tools Collection [13]; and reSearch, comprehensive literature reviews on selected rehabilitation topics which are based on the questions that NARIC specialists received [14]. Other free and downloadable publications include newsletters, reports, and annual NIDILRR Program directories listing current projects.

## HELP

As the physical library of NIDILRR, NARIC's information specialists are available in-person and online to provide free reference and literature search assistance Monday through Friday, during library business hours. While specialists do not provide rehabilitation services, benefits, or financial aid programs, they refer users to specific agencies and other organizations that can provide those services.

A notable feature are the extensive guides (e.g., Frequently Asked Questions (FAQs)) and multiple ways for users to find assistance, tips, search instructions, and other recommendations on its webpages [15]. For example, by clicking on the “help” button in the advanced search screens, users can access searching guides. Most guides connect users to other free recommended resources (e.g., RehabMeasures from NIDILRR). Users can also contact an information specialist via phone, live chat, or by submitting an information request. NARIC has a strong social media presence through its blog, Face-book, Pinterest, and a Twitter account available at the bottom of the webpages.

## ACCESSIBILITY

The NARIC interface has not been recently redesigned [16] and an upgrade would likely give users of this valuable resource a more user-friendly navigation experience. Each of the URLs from NARIC's six tabs were submitted to WAVE: Web Accessibility Evaluation Tool [17]. It will be beneficial to review the color contrast between text and background colors. A review of headings and non-working links is also warranted. Initial navigation of the NARIC interface may be confusing. The webpages share identical color and layout, and some tabs appear crowded with content (Figure 1), making it difficult to identify which webpage or resource is being used. Breadcrumbs are available to mark the location of the webpage, but it would be helpful to see the breadcrumbs in larger font to make them more prominent. In addition, it will help users to access the “Return to top” hyperlink as they navigate the webpages rather than at the bottom of the page. Most guides and supporting documentation are available in HTML and as PDFs.

NARIC should be considered for any search on all aspects of rehabilitation and disability and can serve as a stepping point to additional resources useful to different users, even if the majority of its resources are not available in full text or require a small fee. Researchers can use NARIC as a standalone resource but using other major databases will add to a more comprehensive search because of the interdisciplinary nature of rehabilitation and disability issues, as there are currently no comparable free and specialized databases focusing on rehabilitation and disability.
